# Psychotherapy participants show increased physiological responsiveness to a lab stressor relative to matched controls

**DOI:** 10.3389/fpsyg.2014.00795

**Published:** 2014-07-28

**Authors:** Patrick R. Steffen, Louise Fidalgo, Dominic Schmuck, Yoko Tsui, Tracy Brown

**Affiliations:** Department of Psychology, Brigham Young UniversityProvo, UT, USA

**Keywords:** stress, physiology, psychotherapy

## Abstract

Accumulating evidence indicates that psychotherapy participants show increased physiological responsiveness to stress. The purpose of the present study was to examine differences between individuals participating in outpatient psychotherapy and matched controls using an experimental design. Forty-two psychotherapy participants and 48 matched controls were assessed on cardiovascular and cortisol functioning at baseline, during the Trier Social Stress Test (TSST), and during a 20-min recovery period. Psychotherapy participants and matched controls did not differ at baseline or during the TSST on the physiological measures but psychotherapy participants had higher cortisol and heart rate (HR) during the recovery period. In regards to reactivity, cortisol increased during the recovery period for the psychotherapy participants but decreased for those in the matched control group. Psychotherapy participants experiencing clinically significant levels of distress displayed elevated systolic and diastolic blood pressure and HR during the TSST when compared to psychotherapy participants not experiencing clinically significant levels of distress. Overall, physiological reactivity to stress appears to be an important issue for those in psychotherapy and directly addressing this issue may help improve psychotherapy outcomes.

## INTRODUCTION

High levels of psychological distress contribute to elevated physiological activity and negative health outcomes (Steptoe et al., 2005; Chida and Steptoe, 2010; Carroll et al., 2012). In psychotherapy, high levels of psychological distress are related to elevated physiological activity and addressing difficult topics in therapy such as previous trauma leads to increased physiological responses (Lindauer et al., 2006; Ham and Tronick, 2009; Ehrenthal et al., 2010). This is particularly true in more severe pathology and inpatient studies. Lindauer et al. (2006) in a study of posttraumatic stress disorder found that focusing on trauma cues led to increased physiological reactivity. It is not known however if general psychotherapy participants in outpatient settings have elevated stress physiology relative to non therapy controls. The purpose of the present study was to answer this question using a controlled laboratory stress paradigm comparing psychotherapy participants to a psychotherapy naïve control group.

### THE IMPACT OF STRESS

Stress is a highly prevalent problem with significant negative consequences (American Psychological Association [APA], 2008). In a nationally representative sample of Americans, approximately one third of Americans reported experiencing high levels of chronic stress (Keller et al., 2012). Research has shown that stress has negative effects on physical and mental health, and chronic stress plays a role in the development and progression of physical illness (Rozanski et al., 1999; Krantz and McCeney, 2002; Rosengren et al., 2004; Carroll et al., 2012). One area of physiological health that seems to be particularly affected by stress is cardiovascular disease. Steptoe et al. (2005) found that unemployment and financial difficulties predicted the development of hypertension at a 3-year follow-up, showing that exposure to stress contributes to deterioration of the cardiovascular system.

Stress reactivity, or the way individuals physiologically and emotionally respond to stressful situations, is central in understanding how we are affected by stress and how it impacts our functioning. Physiological measurements, such as cardiovascular indices and hormonal change, are an integral aspect of evaluating individual’s stress reactivity. Increased levels of cortisol have consistently been linked to experienced acute and/or chronic stress (Bohnen et al., 1990; Engert et al., 2012; Aschbacher et al., 2013). Additionally, blood pressure (Juster et al., 2012) and heart rate (HR; Kudielka et al., 2004) are commonly used measures of stress reactivity and recovery. The most commonly used method to assess stress reactivity in the laboratory reported in the research literature is the Trier Social Stress Test (TSST). After establishing baseline levels, participants prepare for an ideal job interview, give the interview, perform math problems, and then rest during a recovery period. The TSST consistently results in elevated blood pressure and cortisol levels and is considered the gold standard of physiological stress reactivity assessment. No studies to date have examined physiological differences between psychotherapy participants and non-therapy controls in their responses to the TSST.

### PSYCHOTHERAPY AND PHYSIOLOGY

Individuals engaged in psychotherapy display exaggerated physiological reactivity to stress. Blanchard et al. (2002) in a study of post traumatic stress disorder (PTSD) found that exposure to trauma cues lead to elevated HR reactivity. They also found that PTSD patients who respond positively to cognitive behavioral therapy displayed decreased HR reactivity in response to those trauma cues. Furthermore, PTSD patients provided with eye movement desensitization and reprocessing therapy have lower skin conductance and lower HR during trauma recall after only one therapy session (Aubert-Khalfa et al., 2008). Similarly, mothers who are clinically depressed show increased stress reactivity during a stress-inducing task compared to non-depressed mothers. One session of interpersonal psychotherapy is effective in significantly decreasing stress reactivity of depressed mothers (Cyranowski et al., 2009).

In a more general study about adolescents’ externalized behavioral problems, Schechter et al. (2012) found that either low or high cortisol levels of adolescents are correlated with negative multisystemic therapy outcome. Additionally, children and adolescents experiencing stress while in treatment for behavioral problems show worse treatment outcome (Mathijssen et al., 1999). However, patients with panic disorder, agoraphobia, or other phobias actually respond better to treatment when they are more reactive to stress during fear inducing situations, as measured by HR (Lang et al., 1998) and cortisol levels (Siegmund et al., 2011).

To determine if stress reduction techniques enhance therapy outcome, Weiss et al. (2005) had participants receive psychotherapy treatment by itself, or stress reducing mindfulness training in addition to psychotherapy. Even though participants in the mindfulness in addition to psychotherapy group did not differ in distress scores after therapy from the psychotherapy group, the former group did have greater goal achievement scores in average. Psychotherapy related improvements in psychological distress have been correlated with decreased stress reactivity (Blanchard et al., 2002; Aubert-Khalfa et al., 2008; Cyranowski et al., 2009). In a pilot study, Ehrenthal et al. (2010) found that physiological stress reactivity prior to treatment predicts therapy outcome in a sample of inpatients hospitalized for depression. Those with low physiological stress reactivity had significantly better psychotherapy outcome compared to patients with high physiological stress reactivity. However, no studies to date have examined physiological stress reactivity in an outpatient setting.

### CURRENT STUDY

The purpose of the present study was to examine whether psychotherapy participants in an outpatient setting would show elevated physiology relative to a matched control group. Three hypotheses were tested. First, psychotherapy participants would show elevated physiology at baseline before beginning the laboratory stressor. Second, psychotherapy participants would show a larger overall response to the laboratory stressor than the control group. And third, the psychotherapy participants would show increased physiological reactivity to the stressor with greater changes from baseline to stressor.

## MATERIALS AND METHODS

### PARTICIPANTS

Forty-two psychotherapy patients were recruited from the Brigham Young University, Provo Utah, counseling center. Psychotherapy patients entering the study had just begun psychotherapy and had received one to two sessions only. A matched control group of 48 college students not receiving psychotherapy were recruited via a research participation system run by the psychology department. Our sample was comprised of college students only. About 57% of participants were females, and 43% were males. The average age of all participants was approximately 23 (SD = 4.1) and the mean BMI was 23.4 (SD = 3.4). This study received Institutional Review Board approval before beginning and all participants read and signed an informed consent form before participating in the study.

### PROCEDURES

#### Overview

The study proceeded in two phases: (a) completion of preliminary questionnaires, and (b) laboratory physiological stress reactivity measurement. All procedures were approved by the Brigham Young University Institutional Review Board. Preliminary questionnaires involved informed consent, a self-report measures of psychological distress, and demographic information. The second phase of the study involved the laboratory stress task. During the laboratory tasks, participants’ physiological measures of stress reactivity were collected. Participants’ completion of the study was compensated with 20 dollars cash.

#### The Trier Social Stress Test

Physiological stress reactivity was assessed through induction of a stressful situation using the TSST (Kirschbaum et al., 1993). The first phase of the TSST involves a baseline rest condition to establish resting blood pressure and levels of cortisol from saliva. During the baseline condition, participants were asked to sit quietly and watch a relaxing video for 15 min. At minutes 11, 13, and 15 of the baseline period BP and HR were measured while and one saliva sample was collected at minute 15. Stress induction occurs during the second phase of the TSST, when participants were asked to prepare for an impromptu speech. Participants were told to prepare mentally for a job interview for their ideal job and to think about how to best present themselves. After 5 min of speech preparation, participants were asked to present their speech in front of an unfamiliar research assistant. They were also told that their speech would be recorded for later analysis by experts. Speech presentation was 5 min. The last phase of the stress indication was a math problem. Participants had to mentally manipulate numbers and solve problems out loud for 5 min. They were told to stop and start over every time they made a mistake. BP and HR were measured at them midpoint and at the end of each task. The last phase of the laboratory tasks was a 20-min rest condition to evaluate how physiological indicators of stress return to normal, with BP, HR, and cortisol being measured at the beginning, midpoint, and end of the recovery period. Presentation of an unexpected speech in the context of evaluation by strangers and mental manipulation of numbers have been shown to significantly increase physiological measures of stress and to provide an accurate representation of an individual’s stress response.

### MEASURES

#### Demographics

Client reported their age and gender, and then were weighed and measured in order to calculate body mass index (BMI).

#### Psychotherapy outcome

Psychotherapy outcome and participant’s progress was monitored using the Outcome-Questionnaire (OQ-45, Lambert et al., 2004). The OQ-45 is a 45-item self-report measure assessing symptom distress, interpersonal relationships, social role, and quality of life in psychotherapy clients (Lambert, 2013). Each item is rated on a 5-point Likert scale ranging from 0-“Never” to 4-“Almost always.” The OQ-45 is a valid and reliable measure of change in clients’ reported distress. Previous research has determined that a cut-off score of 62 is indicative of clinical distress, those receiving mental health treatment typically score above this point, and community samples typically score below this point. It has an excellent internal consistency of 0.93 and a 3-week test-retest reliability of 0.84. In addition, it has a significant concurrent validity with measures of self-report symptoms and psychopathology such as the Beck Depression Inventory (Beck et al., 1996) and the Spielberger State Anxiety Inventory (Spielberger et al., 1983).

### PHYSIOLOGICAL MEASURES

Heart rate, diastolic, and systolic blood pressure (SBP) data were collected using a Dinamap Model 8100 automated blood pressure monitor (Critikon Corporation, Tampa, FL, USA) that capitalizes on the oscillometric method. Readings were obtained following the specifications of the manufacturer using a cuff that was measured and properly sized to fit on the upper non-dominant arm of the participant. Cortisol was measured via saliva samples. Salivary samples were stored at -20°C until analysis. After thawing the samples, the salivettes were centrifuged for 5 min at 3000 rpm. Concentrations of salivary cortisol were measured using a commercially available immunoassay with chemiluminescence detection (CLIA; IBL, Hamburg, Germany).

### DATA ANALYSIS

Before analyzing the research questions, experimental groups were first compared to examine whether groups were not significantly different at baseline for demographic, blood pressure, and psychological distress using independent sample *t*-tests and *chi*-square analyses. 2-Group × 7-Time repeated measures analyses of variance (ANOVAs) were used to analyze the research questions. We report partial-eta2 ($\eta_{p}^{2}$) for ANOVA effect sizes and significant main effects and interactions were decomposed using follow-up contrasts. Main effects for time were calculated to examine the impact of the experiment on blood pressure and HR from baseline to recovery, including the stressor. Time main effects were followed up by analyses of Group × Time interactions and tests of group differences.

## RESULTS

### SAMPLE CHARACTERISTICS

**Table 1** displays the sample characteristics by experimental group. The psychotherapy and control groups were not significantly different on gender composition, age, or BMI. Similarly, there were no differences in baseline physiology between groups. The psychotherapy group scored significantly higher on the Outcome Questionnaire, a measure psychological distress that is related to psychotherapy outcome [Psychotherapy mean = 73.6 (25.7) and control group mean = 43.1 (16.5), *t* = 4.280, *p* < 0.001]. Gender and BMI were related to blood pressure and HR such that men had higher SBP (*t* = 6.073, *p* < 0.001) and lower HR (*t* = -3.230, *p* < 0.01) and those with higher BMI had higher SBP (*t* = 4.139, *p* < 0.001). Therefore, gender and BMI were used as covariates in the following analyses because of their significant relationship with the physiological measures.

**Table 1:** Sample characteristics by experimental group.

	Psychotherapy group (*n* = 42)	Control group (*n* = 48)	*p*
**Demographics and distress**
Gender (% female)	57%	56%	0.93
Age	22.9 (4.1)	23.0 (4.4)	0.88
Body mass index	23.4 (3.4)	24.3 (5.2)	0.32
Outcome questionnaire	73.6 (25.7)	43.1 (16.5)	<0.001
**Baseline physiology**
Systolic blood pressure (mm/Hg)	108 (10)	109 (11)	0.49
Diastolic blood pressure (mm/Hg)	65 (7)	64 (7)	0.71
Heart rate (bpm)	69 (11)	73 (11)	0.10
Cortisol (nmol/l)	11.8 (7.4)	11.4 (7.1)	0.80
**Recovery physiology**
Systolic blood pressure (mm/Hg)	112 (13)	112 (12)	0.85
Diastolic blood pressure (mm/Hg)	67 (7)	65 (7)	0.37
Heart rate (bpm)	74 (11)	70 (11)	0.03
Cortisol (nmol/l)	12.7 (10.9)	9.5 (4.9)	0.01

### BASELINE PHYSIOLOGY AND AVERAGE PHYSIOLOGICAL STRESS RESPONSE

The psychotherapy group and matched control group did not differ at baseline on measures of SBP, diastolic blood pressure (DBP), HR, or cortisol. In other words, participants in psychotherapy were not more physiologically aroused than matched controls at the beginning of the experiment. Similarly, there were no differences in average physiological stress responses between groups. During the beginning of the recovery phase, those in the psychotherapy group had elevated cortisol [*F*(1,89) = 7.448, *p* < 0.01, $\eta_{p}^{2}$ = 0.07] and HR [*F*(1,88) = 4.635, *p* < 0.05, $\eta_{p}^{2}$ = 0.05] relative to the matched control group (see **Table 1**). When examining only those in psychotherapy by level of clinical distress (measured by the Outcome Questionnaire, a score above 62), there were significant differences in average physiological response (see **Table 2**). Psychotherapy participants with high levels of distress displayed larger average physiological stress responses for SBP [*F*(1,36) = 4.923, *p* < 0.05, $\eta_{p}^{2}$ = 0.12], DBP [*F*(1,36) = 6.280, *p* < 0.05, $\eta_{p}^{2}$ = 0.15], and HR [*F*(1,36) = 5.017, *p* < 0.05, $\eta_{p}^{2}$ = 0.12].

**Table 2:** Sample characteristics by level of clinical distress for those in the psychotherapy group only.

	High distress (*n* = 28)	Low distress (*n* = 12)	*p*
**Demographics and distress**
Gender (% female)	58%	56%	0.90
Age	23.2 (4.5)	22.8 (4.1)	0.60
Body mass index	23.1 (3.7)	24.3 (4.8)	0.20
**Overall physiology (average across all tasks)**
Systolic blood pressure (mm/Hg)	117 (10)	110 (11)	0.03
Diastolic blood pressure (mm/Hg)	66 (7)	62 (7)	0.02
Heart rate (bpm)	76 (12)	69 (10)	0.03
**Baseline physiology**
Systolic blood pressure (mm/Hg)	109 (10)	108 (11)	0.94
Diastolic blood pressure (mm/Hg)	66 (7)	64 (7)	0.19
Heart rate (bpm)	72 (12)	70 (10)	0.40
**Physiological stress reactivity (math)**
Systolic blood pressure (mm/Hg)	126 (16)	123 (15)	0.082
Diastolic blood pressure (mm/Hg)	77 (9)	73 (8)	0.395
Heart rate (bpm)	81 (15)	80 (12)	0.757

### PHYSIOLOGICAL REACTIVITY TO THE LABORATORY STRESSOR

**FIGURE 1:**
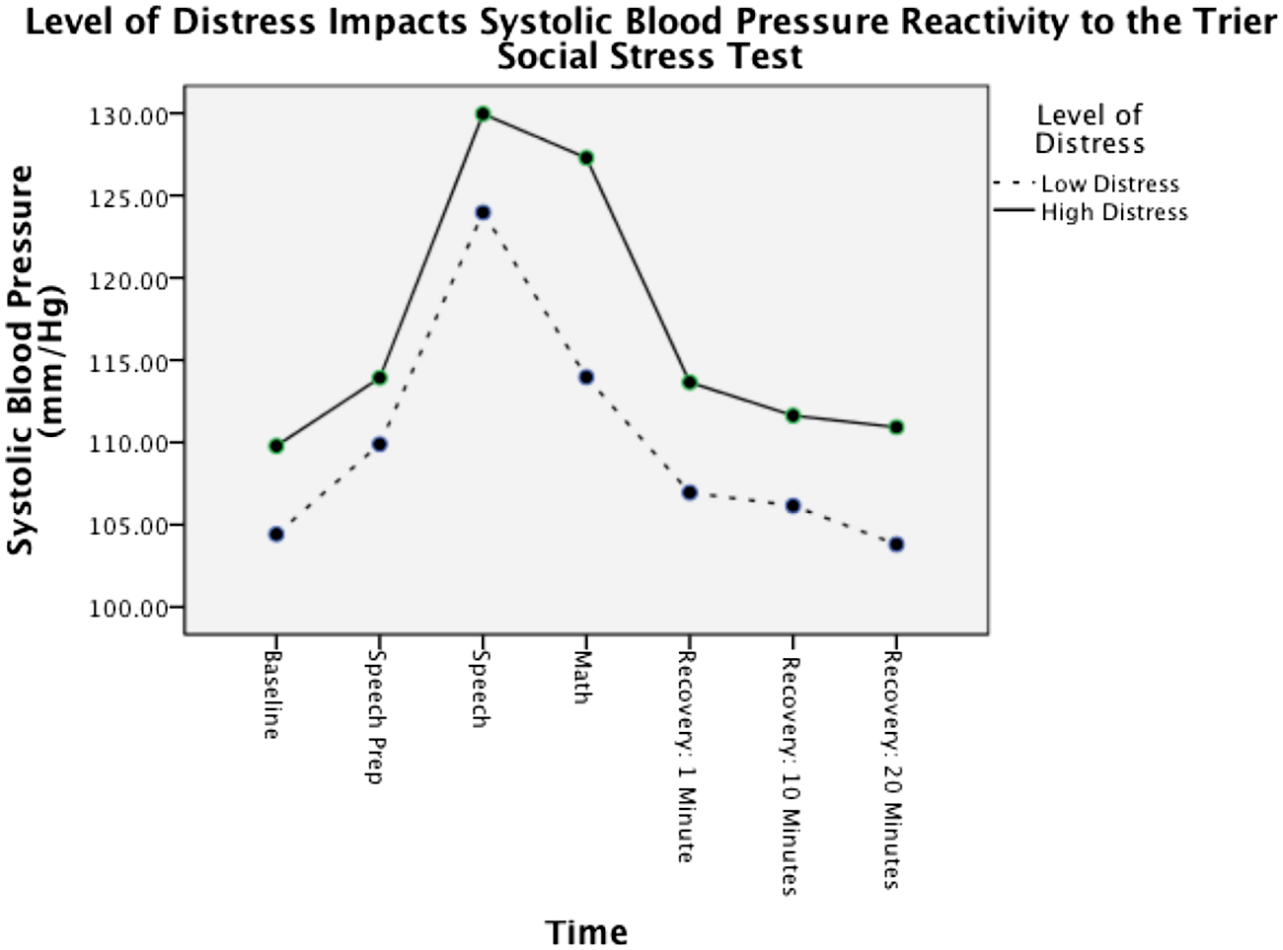
**Psychotherapy participants with high levels of clinical distress (an Outcome Questionnaire score above 62) show higher levels of blood pressure reactivity to the Trier Social Stress Test**.

Because cortisol was not different between groups at baseline but did differ during recovery, there was a significant difference in terms of physiological reactivity following the stressor. The overall within subjects analysis was *F*(3,282) = 5.471, *p* < 0.01, $\eta_{p}^{2}$ = 0.06 indicating different response patterns for the psychotherapy and matched control groups. Specifically, the psychotherapy participants displayed an increase in cortisol following the presentation of the laboratory stressor whereas the control group trended lower [*F*(1,94) = 6.749, *p* < 0.05, $\eta_{p}^{2}$ = 0.07; see **Table 1**]. For SBP, there was a non significant trend for those clinically distressed to have more physiological reactivity [*F*(1,210) = 4.923, *p* = 0.082, $\eta_{p}^{2}$ = 0.050; see **Table 2**]. This difference was most strongly seen during the math portion of the laboratory stressor with the clinically distressed participants in the psychotherapy group still showing relatively high levels of SBP, whereas the non-clinically distressed participants showed a significant drop in SBP [*F*(1,36) = 4.083, *p* = 0.05, $\eta_{p}^{2}$ = 0.10; see **Figure 1**].

## DISCUSSION

The purpose of this study was to examine whether physiological response to a laboratory stressor would be higher in psychotherapy participants relative to a matched control group. It was hypothesized that psychotherapy participants would have elevated baseline physiology, elevated average physiological response, and greater reactivity to a speech and math stressor relative to baseline levels. No group differences in physiology were found at baseline. Those in psychotherapy did report higher levels of psychological distress as might be expected. There were no differences between groups in average physiological response; however there was a significant difference when comparing those with high levels of clinical distress with those with low levels. Those high in clinical distress displayed higher overall SBP, DBP, and HR to the TSST than did the low distress group. In regards to physiological reactivity, the psychotherapy group showed greater cortisol levels following the TSST relative to the control group indicating a stronger stress response.

There are three key implications of these findings. First, not everyone engaged in psychotherapy has clinical levels of distress and it appears that overall level of clinical distress is an important factor in physiological response to stress. However, most of those who qualified as clinically distressed were in the psychotherapy group. Given the limited size of this study and the small number of controls with clinical distress, there was insufficient power to see whether there was an interaction between psychotherapy participation and clinical distress.

The second implication is that psychotherapy participants may look normal physiologically at rest but have an exaggerated response to stressful situations. To examine the impact of stress in psychotherapy, it is insufficient to measure just baseline physiology. Rather, it is the reaction to and recovery from stressful situations that are important (Zautra, 2009). This finding is in line with Heim et al. (2000) who found that those with depression and trauma did not appear different physiologically at baseline but had a large physiological stress response relative to controls. Resilience may play a key role here (see Connor and Davidson, 2003; Zautra, 2009). Those higher in resilience may handle stress more effectively and thereby recover from stress more quickly. It is also possible that successful psychotherapy increases resilience and thereby decreases physiological reactivity to stress (Lane et al., 2009; Thayer et al., 2012).

The third implication is that stress reduction strategies may be a useful adjunct for those in psychotherapy. High levels of stress can interfere with attention and focus and stress reduction may help improve psychotherapeutic efforts by reducing physiological stress symptoms. Marci and Riess (2005) and Riess (2011) argue for the clinical relevance of using psychophysiologic measures as an important adjunct in psychotherapy that has the potential to improve therapy quality and help guide therapeutic decisions. It is also possible that the current focus on mindfulness approaches in cognitive behavioral psychotherapy is at least partially driven by its success in reducing stress. Additionally, HRV biofeedback has shown promise as an adjunctive treatment in depression and anxiety, with stress reduction likely playing a key role in this affect (Karavidas et al., 2007; Lehrer and Eddie, 2013).

There are several limitations to this study. First, this study was cross sectional in nature so it is not clear how stress physiology impacts long term outcome in psychotherapy. Second, the sample consisted of relatively young, healthy college students with psychotherapy being administered in a counseling center. It is not known if these results will generalize to other age groups, conditions, or different clinical settings. Strengths of the current study were the inclusion of a matched control group with no previous psychotherapy experience and a controlled experimental design. Future studies could build on these findings by conducting controlled experimental longitudinal studies to examine how reducing stress physiology is related to psychotherapy outcome and by looking at different age groups and different clinical settings. Additionally, focusing on the role of resilience in the relationship between psychotherapy and psychophysiological stress reactivity may be especially fruitful (see Connor and Davidson, 2003). It is possible that psychotherapy increases resilience in the face of significant stress (Lane et al., 2009; Thayer et al., 2012).

In conclusion, people with who are clinically distressed display greater physiological response and greater physiological reactivity to a laboratory stressor relative to a matched control group. Distressed individuals did not differ at baseline physiologically indicating resting physiological measures may be insufficient to identify those who may be at risk of stress related problems in psychotherapy. Rather, examining stress response as well as ability to recover from stress once the stressor is over is crucial. Stress reduction techniques may be a beneficial adjunct to psychotherapy and future studies could examine this possibility using a longitudinal controlled experiment.

## Conflict of Interest Statement

The authors declare that the research was conducted in the absence of any commercial or financial relationships that could be construed as a potential conflict of interest.
